# Distributed power analysis attack on SM4 encryption chip

**DOI:** 10.1038/s41598-023-50220-2

**Published:** 2024-01-10

**Authors:** Haoran Gong, Tailiang Ju

**Affiliations:** 1 UESTC, No. 2006 Xiyuan Avenue, Chengdu, 611730 Sichuan China; 2 SICE, UESTC, No. 2006, Xiyuan Ave, West Hi-Tech Zone, 611731 Chengdu, China

**Keywords:** Computer science, Information technology

## Abstract

Encryption chips are specialized integrated circuits that incorporate encryption algorithms for data encryption and decryption, ensuring data confidentiality and security. In China, the domestic SM4 algorithm is commonly utilized, as opposed to the international AES encryption algorithm. These widely implemented encryption standards have been proven to be difficult to crack through crypt analysis methods Currently, power consumption side-channel attacks are the most prevalent method. They involve capturing power consumption data during the encryption process and subsequently recovering the encryption key from this data. The two leading methods are Differential Power Analysis (DPA) and machine learning techniques. DPA does not necessitate prior knowledge but relies heavily on the number of power consumption curves. With only 50 power consumption data points, the accuracy is a mere 80%. Machine learning methods require prior knowledge, achieving an accuracy rate above 95% with only 30 power traces, albeit with training times typically exceeding 15 min. In this paper, a distributed energy analysis attack approach was presented based on Correlation Power Analysis (CPA). The power consumption data was divided into 16 subsets, with each subset corresponding to 8 bytes of the key. By training each subset separately, the 8-byte key’s corresponding power consumption data is reduced to only 100 dimensions, resulting in a 76% decrease in cracking time and a 3% improvement in cracking accuracy rate.This article also trains a more complex 256 classification model to directly crack the final key, achieving a success rate of 28% in cracking 128-bit passwords with only 1 power trace

## Introduction

Encryption chips play a significant role in today’s society, in areas such as financial payments, smart cards, mobile devices, and more. For example, many electronic devices like bank cards, smartphones, and electronic passports use secure chips to store and protect sensitive information. However, despite employing various encryption algorithms to safeguard data security, they are not always secure in practice. The compromise of encryption chips can lead to various harms, including financial losses, personal privacy breaches, security vulnerabilities, and more.

Side-channel attacks and power analysis attacks have become hot topics in the field of encryption chip attacks. Side-channel attacks are a method of attack that can decrypt keys by monitoring the electromagnetic radiation, power consumption, or other physical characteristics generated by encryption chips. However, side-channel attacks are not always feasible because they require significant computation and analysis, along with physical access to the hardware of the encryption chip. With technological advancements, power analysis attacks have also become a potent weapon for decrypting encryption chips. This attack method has seen rapid development over the past few years, and many research findings related to power analysis attacks have been published in international journals and conferences.

In 1999, KOCHER proposed the power side-channel attack method^1^, which provided an alternative encryption attack method aside from mathematical analysis. This method revealed the relationship between encryption hardware and encrypted data by using physical information generated during hardware data encryption, such as power consumption, electromagnetic radiation, and time-based data, to decrypt encryption algorithms^2^. Subsequently, power analysis attacks have been widely applied and developed. For example, Chari et al. and Mangard et al. proposed attack methods based on power and electromagnetic radiation analysis, referred to as ’Differential Power Analysis’ and ’Differential Electromagnetic Analysis,’ respectively. Additionally, with the rapid development of machine learning, especially deep learning^3^, there is an increasing number of researchers applying machine learning to side-channel attacks, and its effectiveness far surpasses traditional analytical methods. Backs et al^4^. applied machine learning to sound side-channel attacks on printers, Hospodar et al^5^. classified intermediate values in template attacks using least squares support vector machines, Lerman et al^6^. used algorithms like random forests, support vector machines, self-organizing maps for side-channel analysis, Heuser et al^7^. employed multi-class support vector machines for attacking multi-value (Hamming weight models), Bartkewitz et al^8^. further improved the aforementioned work, proposing new multi-classification strategies based on categories, and Martinasek et al.citebib9. introduced a neural network-based AES side-channel attack method and classified AES keys.

Unlike the commonly used international Advanced Encryption Standard (AES), the SM4 encryption algorithm is an emerging algorithm and is one of the commercial cryptographic algorithms recommended by the China National Cryptography Administration. The design goal of the SM4 algorithm is to provide high-strength data encryption protection while ensuring sufficient security, efficiency, and flexibility.

The use of the SM4 algorithm is widespread, especially in fields such as mobile payments, the Internet of Things (IoT), and cloud computing. As a cryptographic algorithm developed independently by China, the SM4 algorithm has gained broad international recognition and application. In addition to its extensive use in areas such as finance and government, the SM4 algorithm has also been approved by the International Organization for Standardization (ISO/IEC) as an international standard, receiving widespread international adoption.

Compared to the AES algorithm, the SM4 algorithm is more resistant to attacks such as power analysis. The interaction between the key and plaintext in SM4 is more concealed. Additionally, the key decryption in SM4 is not independent. If the higher-order bits of the key are decrypted incorrectly, it will inevitably lead to errors in the lower-order bits of the key. This characteristic makes the success rate of breaking SM4 consistently lower than that of AES. Moreover, the training models for machine learning in the context of SM4 are also more complex.

The aim of this work is to minimize the decryption time and reduce the number of power traces used, while ensuring a successful decryption rate.

## SM4 encryption algorithm and hardware implementation

### SM4 encryption algorithm

The SM4 algorithm employs a 128-bit key and has a block key length of 128 bits. It’s processes include round key addition, S-Box substitution, linear transformation, key expansion, and inverse operations.

**Figure 1:**
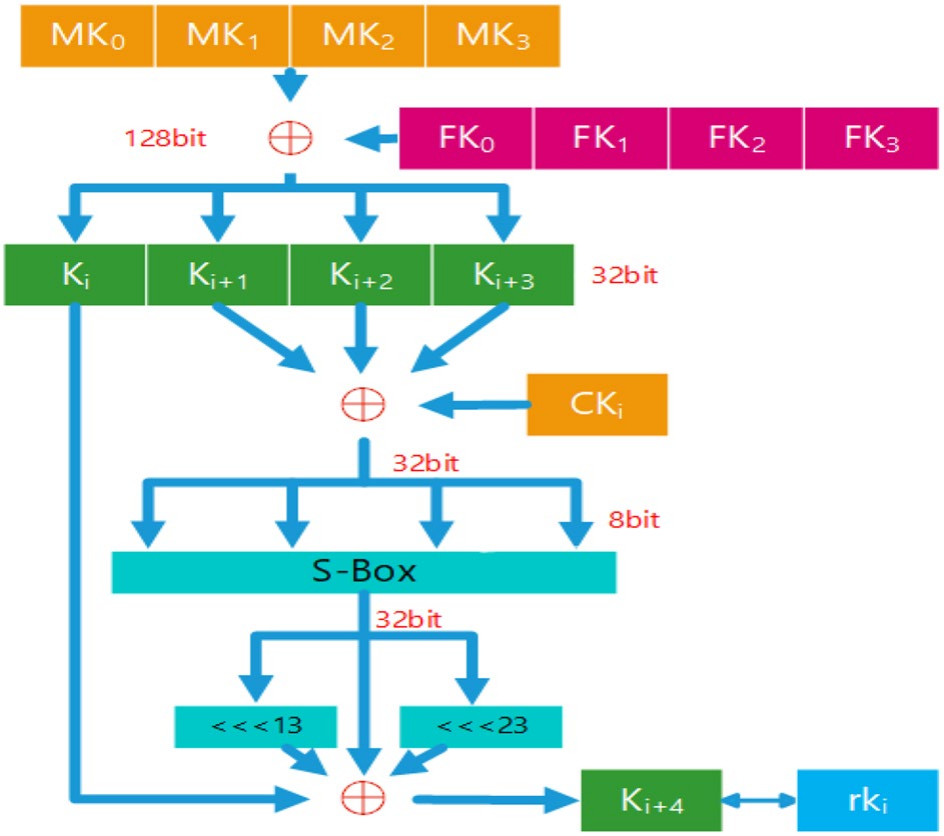
SM4 key expansion.

**Figure 2:**
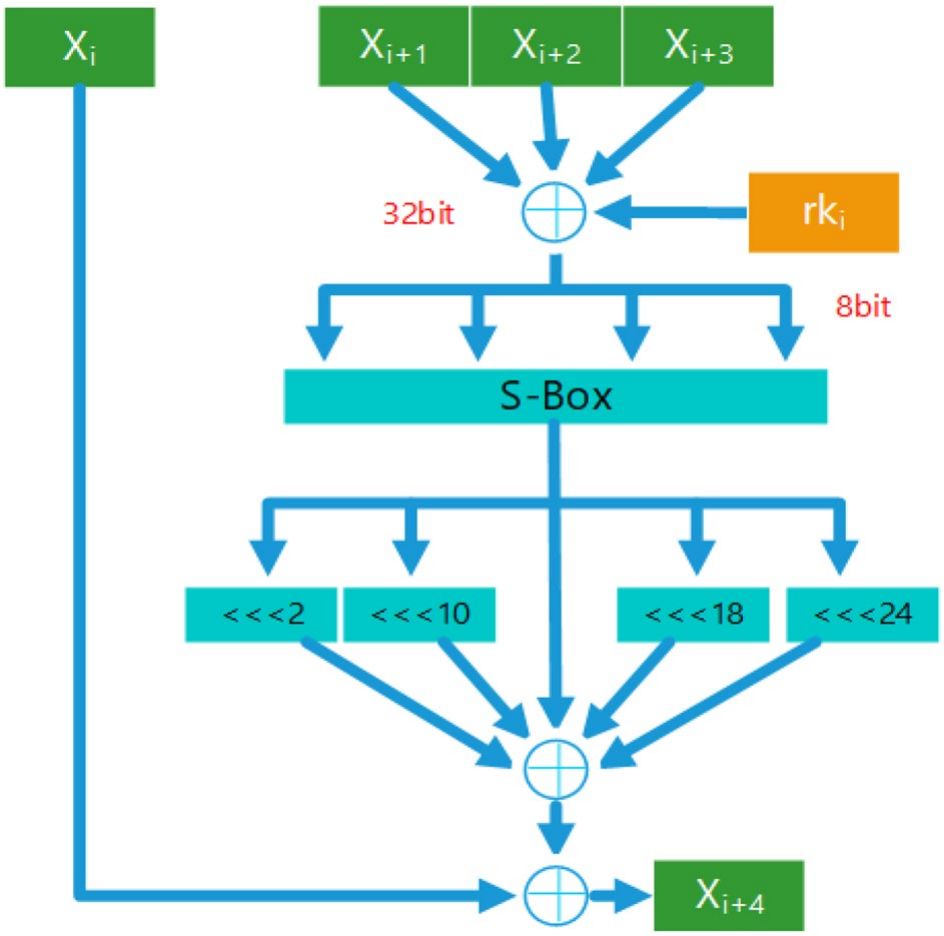
SM4 encryption process.

The first step is key expansion as Fig. 1, which extends the key *MK* into 32 round keys $rk_i$:

The second part is plaintext encryption as Fig. 2:

The entire encryption process can be described using a simple formula:

Where the key *MK*, plaintext *PT*, and ciphertext *CT* are all 128 bits, and the key $MK_i$, $X_i$, and $rk_i$ are all 32 bits.

1 $$\begin{aligned} MK&=[MK_0,MK_1,MK_2,MK_3] \end{aligned}$$

2 $$\begin{aligned} PT&=[X_0,X_1,X_2,X_3] \end{aligned}$$

3 $$\begin{aligned} F_1(MK)&=[rk_0,rk_1,rk_2....rk_{31}] \end{aligned}$$

4 $$\begin{aligned} F_2(X_0,X_1,X_2,X_3,rk_0)&=X_4\nonumber \\ F_2(X_i,X_{i+1},X_{i+2},X_{i+3},rk_i)&=X_{i+4}\nonumber \\ ..........................&\nonumber \\ F_2(X_{31},X_{32},X_{33},X_{34},rk_{31})&=X_{35} \end{aligned}$$

5 $$\begin{aligned} CT&=[X_{35},X_{34},X_{33},X_{31}] \end{aligned}$$

### Hardware implementation of SM4 encryption

The chip chosen for this project is the Atmel Xmega-128D4, and the target board used is the ChipWhisperer CW308 UFO board.

ChipWhisperer is a company specialized in providing side-channel attack tools and training. CW308 is one of their produced target boards used for side-channel attack experiments.

The CW308 target board features a 50 MHz XMEGA microcontroller and provides various peripheral interfaces, such as a high-speed ADC (12-bit, 105 MSPS), programmable clock (100–500 MHz), and a USB interface for connecting to the host computer. This board can be used in conjunction with the ChipWhisperer Lite or Pro USB analyzer for the analysis and attack of side-channels in embedded systems.

The hardware implementation steps for the SM4 encryption algorithm are as follows:

1. Microchip Studio is selected as the development environment, and the C++ code for SM4 encryption is initially written.
2. The project’s main program, serial communication program, and driver programs are completed and compiled.
3. The generated HEX file is burned into the Xmega128D4 chip.

## Collection of SM4 encryption power traces

**Figure 3:**
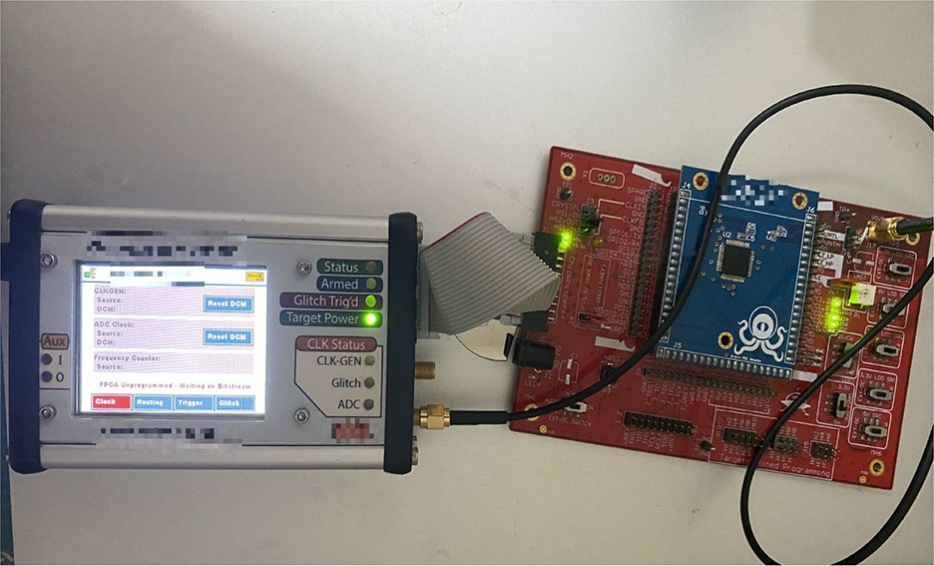
Power consumption acquisition platform.

ChipWhisperer CW1200 Power Consumption Acquisition Platform(Fig. 3)

### Principle of power traces collection

The CPU exhibits different power consumption characteristics when executing different instructions. This is because different instructions trigger different numbers of semiconductors, and some instructions may access memory, cache, and so on. Complex instructions may also require more clock cycles than others, leading to various factors that result in distinct power consumption patterns during instruction execution. ChipWhisperer, through its power measurement interface, can detect voltage variations on the VCC power line of the target chip. The greater the decrease in voltage, the higher the current CPU power consumption. By accurately sampling power variations, we can create a graph illustrating the changes in CPU power consumption. This allows us to identify relevant signal features that leak information about CPU operations and subsequently exploit them.

### The settings for power consumption data acquisition

**Figure 4:**
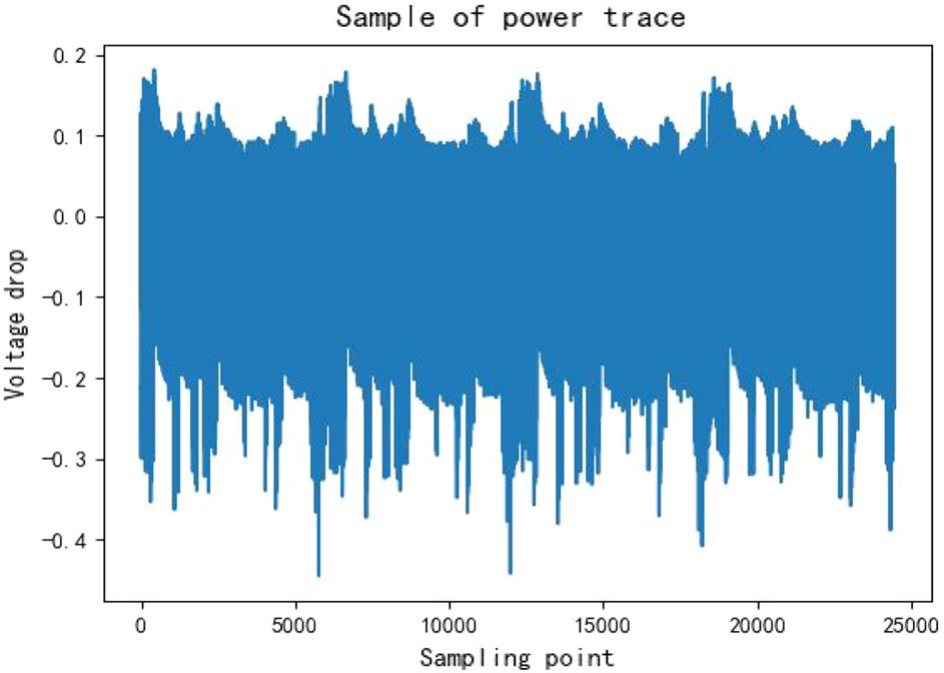
Examples of power traces (Power Consumption Curves).

In this study, the number of sampling points is set at 24,400, with an analog-to-digital converter offset of 1250, and triggering on the rising edge. Two different power consumption curves are collected:

1. 500 power consumption traces with a fixed key and random plaintext, used for decryption.
2. 10,000 power consumption traces with random key and random plaintext, used for training in machine learning methods.

* Figure 4 is an example of power trace

## Power analysis attack

### Correlation power analysis attack (CPA)

#### Attack principle

The effectiveness of side-channel attack depends on the selection of attack points within the cryptographic algorithm and the corresponding energy model.

In the case of the SM4 encryption algorithm, during each round iteration, the input for each round is XORed with the round key for that round, followed by passing through an S-box. The S-box is a non-linear transformation that generates significant power consumption during the transformation, as compared to linear transformations. Therefore, choosing this point as an intermediate value for power analysis attacks makes it easier to break the key. This value, denoted as $V_{atk}^i$, can be represented as follows:

6 $$\begin{aligned} V_{atk}^i=HW(Sbox(X_{i+1}\oplus X_{i+2}\oplus X_{i+3}\oplus rk_{i} )) \end{aligned}$$

* *HW*() converts numbers into Hamming weight.

#### Cracking of the round key

7 $$\begin{aligned} rk_0=[rk_0[0],rk_0[1],rk_0[2],rk_0[3]] \end{aligned}$$

Starting with the cracking of $rk_0[0]$, proceed to crack every 8 bits:

(1) With *n* identical keys and different plaintexts, compute the correlation coefficient between the power trace samples and the intermediate value Hamming weight, as expressed by the following formula:

$P_{jk}$ represents the value of the *k*-th sample point of the *j*-th power trace, and $P_{k}$ represents the value vector of all power traces at time *k*:”

8 $$\begin{aligned} P_{k}=[P_{0k},P_{1k},P_{2k},.....P_{nk}] \end{aligned}$$

(2) For each power trace, calculate *V* for $m\in (0,256)$:

9 $$\begin{aligned} PT^j=[X_0^j,X_1^j,X_2^j,X_3^j] \end{aligned}$$

$PT^j$ represents the plaintext of the *j*-th power trace.

10 $$\begin{aligned} X_{l}^j=&[X_{l}^j[0],X_{l}^j[1],X_{l}^j[2],X_{l}^j[3]] \end{aligned}$$

11 $$\begin{aligned} V_{rk_0[0]=m}^j=&HW(Sbox(X_{1}^j[0]\oplus X_{2}^j[0]\oplus X_{3}^j[0]\oplus m)) \end{aligned}$$

12 $$\begin{aligned} V_{rk_0[0]=m}=&([V^0,V^1......V^n]|rk_0[0]=m) \end{aligned}$$

(2) Calculate the correlation coefficient *Cor*(*k*, *m*).

13 $$\begin{aligned} Cor(k,m)=Cor(P_k, V_{rk_0[0]=m}) \end{aligned}$$

Where $P_k$ and $V_{rk_0[0]=m}$ are both n-dimensional vectors, and their correlation coefficient calculation formula is:

14 $$\begin{aligned} Cor(P,V)=\frac{\sum _{i=1}^{n}\left( P_{i}-\bar{P}\right) \left( V_{i}-\bar{V}\right) }{\sqrt{\sum _{i=1}^{n}\left( P_{i}-\bar{P}\right) ^{2}} \sqrt{\sum _{i=1}^{n}\left( V_{i}-\bar{V}\right) ^{2}}} \end{aligned}$$

Iterate through sample points $k\in (0,24400)$ and $m\in (0,256)$, searching for the maximum point. At this point:

15 $$\begin{aligned} MAX(Cor(P_k,V_{rk_0[0]=m}))=Cor(P_{pos},V_{rk_0[0]=key}) \end{aligned}$$

Take *key* as the cracked value for $rk_0[0]$, it appears at the *pos*-th sample point in the power trace. Use this method to sequentially crack $rk_0[1]$, $rk_0[2]$, $rk_0[3]$, and obtain the complete $rk_0$.

(3) Iterate 3 times to crack $rk_{1}$, $rk_{2}$, and $rk_{3}$.

16 $$\begin{aligned}&F_2(X_0,X_1,X_2,X_3,rk_0)=X_4 \end{aligned}$$

17 $$\begin{aligned}&V_{atk}^1=Sbox(X_2\oplus X_3\oplus X_4\oplus rk_1 ) \end{aligned}$$

Equation (17) is used to crack $rk_1$. In total, 4 rounds of cracking are performed to obtain $rk_{0}$, $rk_{1}$, $rk_{2}$, and $rk_{3}$, from which the original key is reconstructed.

#### Recovery of key

Given $rk_{0}$, $rk_{1}$, $rk_{2}$, $rk_{3}$, with $CK_i$ as fixed parameters, and $L'$ representing a constant linear transformation within the encryption, the formula yields:

18 $$\begin{aligned} K_i=rk_i\oplus L'(Sbox(rk_{i-3}\oplus rk_{i-2}\oplus rk_{i-1}\oplus CK_i)) \end{aligned}$$

Subsequently, the SM4 key *MK* is reconstructed from $K_0$, $K_1$, $K_2$, $K_3$, and $FK_i$ (fixed parameters), following this computation method:

19 $$\begin{aligned} MK_i&=K_i\oplus FK_i \end{aligned}$$

20 $$\begin{aligned} MK&=[MK_0,MK_1,MK_2,MK_2] \end{aligned}$$

**Figure 5:**
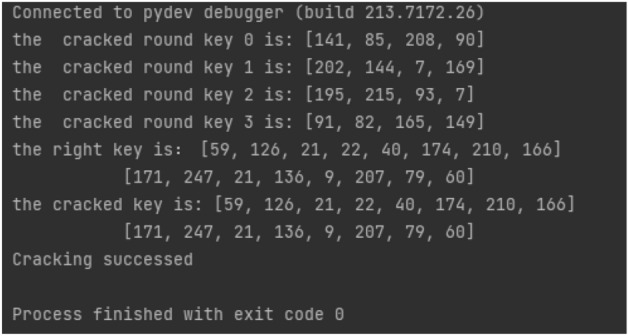
Successful CPA attack.

At this point, the initial key is fully recovered (Fig. 5).

#### The weaknesses of CPA attack

**Figure 6:**
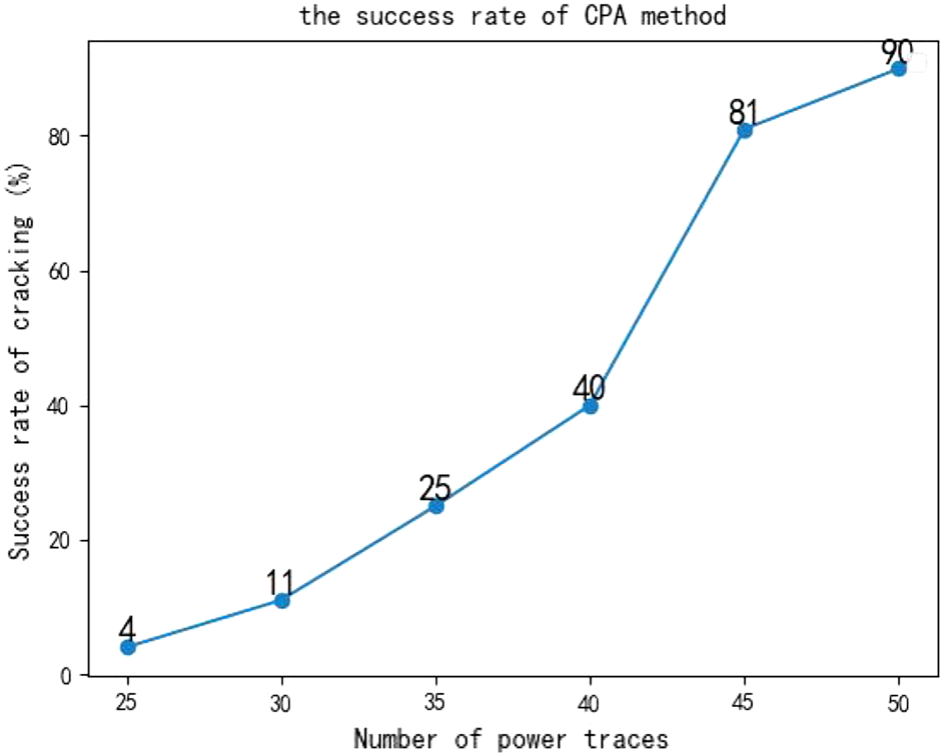
Cracking success rate of CPA method.

1. CPA attacks depend on the number of power traces to be cracked. In the experiments, the number of power traces was gradually reduced, and multiple inputs were used. The success rate of CPA attacks on the SM4 encryption chip is as Fig. 6:
2. The CPA method relies on clock alignment, and it cannot crack the key when clock asynchrony is introduced.
3. The CPA method cannot crack the key when random masking is applied.

### Machine learning-based power analysis attack

#### Attack principle

For each power trace, there is a corresponding attack intermediate value. If multiple power traces are obtained with random plaintext and random keys, and their corresponding intermediate values are calculated, with power traces represented as $\vec {X}$ and the Hamming weight of the corresponding intermediate value as *Y*, we can use machine learning methods to train a model:

21 $$\begin{aligned} f(\vec {X})=Y \end{aligned}$$

Then, the trained model is applied to the target power traces for key recovery^10^.

#### Attack steps

(1) PCA (Principal Component Analysis) Dimensionality Reduction

The original power traces consist of 24,400 sampling points. Regardless of the training mode, a dataset with 24,400 dimensions would require an impractically long training time. The core idea of PCA is as follows: the principal components of a matrix are the eigenvectors of its covariance matrix, sorted by their corresponding eigenvalues. PCA reduces a set of potentially correlated high-dimensional variables into a set of lower-dimensional, linearly uncorrelated variables known as principal components. These lower-dimensional data components aim to retain as much of the original data’s variance as possible. Without delving into specific details, the PCA algorithm can be applied to achieve data dimensionality reduction through the use of PCA API calls.

(2) Machine Learning-Based Key Recovery

Using machine learning methods, a corresponding *Y* is obtained for each power trace.

22 $$\begin{aligned}{}&f(\vec {X_i})=Y_i \\ \vec Y=&[Y_0,Y_1......Y_n] \end{aligned}$$

Taking the attack on $rk_0[0]$ as an example, iterate through $rk_0[0]\in (0,256)$, corresponding to the *i*-th power trace, with the intermediate value:

23 $$\begin{aligned} \begin{aligned} (V_i|rk_0[0]=m)=Y_i^m \\ \vec {Y^m}=[Y_0^m,Y_1^m......Y_n^m] \end{aligned} \end{aligned}$$

Compare each bit of all $\vec {Y^m}$ with $\vec Y$ one by one, and determine the value of *m* that makes the most identical bits as the cracked key.

#### Attack performance

We trained with 10,000 power traces and used 30 power traces for key cracking. We employed three different methods: SVM, LSTM, and CNN. Multiple experiments were conducted, and the results are recorded as follows:

**Table 1:** Cracking speed and success rate of various machine learning methods.

Dims(PCA)	2000	1600	1200	800	400
SVM(S)	968	831	782	704	646
LSTM(S)	1261	1140	1036	920	840
CNN(S)	1128	1045	898	832	785
SVM(%)	95	90	72	66	51
LSTM(%)	97	95	78	70	65
CNN(%)	97	92	75	73	57

$^{1}$ (s) represents the cracking time of the method.

$^{2}$ (%) represents the success rate of the cracking

From Table 1, it can be observed that as the dimensionality increases, the training time becomes longer, and the success rate improves. Conversely, with fewer dimensions, training time is reduced, but the success rate decreases.

Analyzing the underlying reasons, excessive dimensionality reduction results in data loss. While it may improve speed, it leads to a decrease in success rate. PCA dimensionality reduction employs the same approach for cracking each round key, which makes it unable to capture the specific sampling points corresponding to each round key, preventing precise matching.

The appendix includes SM4 power traces and a self-made SVM-based SM4 encryption chip decryptor, with customizable parameters.

#### Comparison between the CPA method and the machine learning method

CPA method and machine learning method have their own advantages and disadvantages. CPA method does not require prior information and has a fast cracking speed, but it requires a large number of power traces to be cracked. In contrast, the machine learning cracking method requires prior information (a significant amount of historical power traces), has a slower cracking speed, but requires fewer power traces to be cracked.Their characteristics are shown in Table 2.

**Table 2:** Comparison of CPA and machine learning.

Cracking Method	Prior information	Cracking speed	Sucess rate (traces used)			
			10	25	40	55
CPA	Not required	Within 1 minute	0%	4%	40%	92%
Machine learning	Need historical power traces	Several minite to train	87%	95%	99%	100%

## Distributed power analysis attack

### Attack principle

Distributed power attack is based on the correlation coefficient between each 8-bit round key and the power trace, which samples the power trace to generate 16 sub-power traces. This reduces the dimensionality of the data, thereby improving the cracking efficiency. At the same time, each sub-power trace is more targeted and less susceptible to interference, which can increase the success rate.

**Figure 7:**
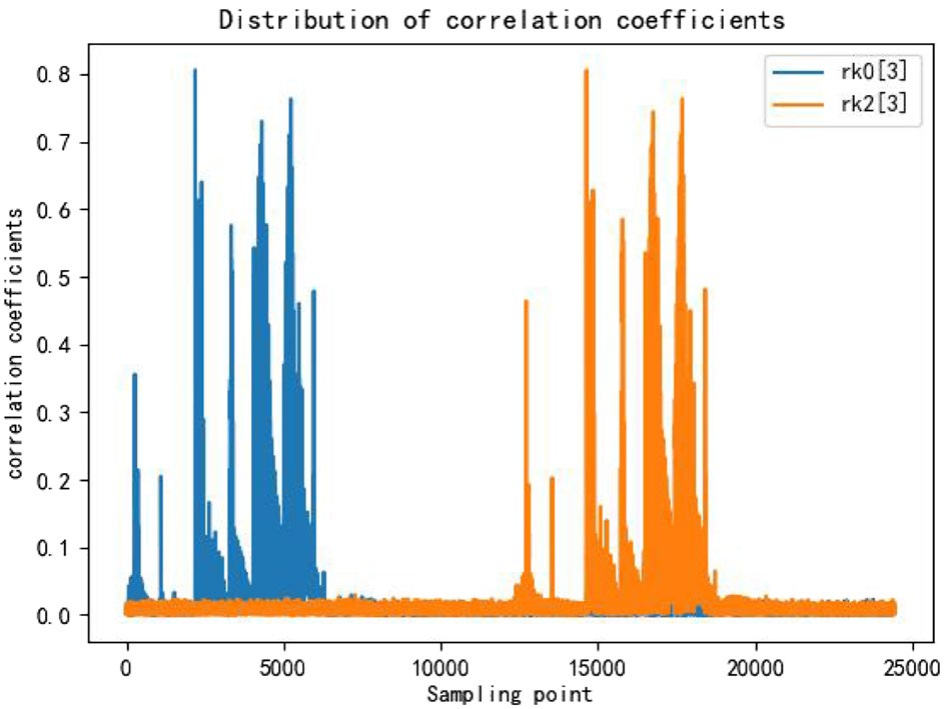
The correlation coefficient of rk0[3] and rk2[3].

From Fig. 7, it can be observed that different round keys manifest at different positions on the power traces. By considering the magnitude of the correlation coefficients, it is possible to extract the sampling points from the power traces for each round key.

### Attack performance

In this study, we selected the top 100 points with the highest correlation coefficients for extraction, forming 16 sub-traces. We then trained them separately using machine learning-based methods and conducted multiple experiments, with the results as follows:

**Table 3:** Success rates of various machine learning methods.

Power trace	30	25	20	15	10
SVM(%)	96	93	87	79	72
LSTM(%)	98	95	90	85	77
CNN(%)	96	92	84	71	65

**Table 4:** Processing times of various machine learning methods.

SVM	LSTM	CNN
190S	370S	235 s

From Tables 3 and 4, it can be observed that the three machine learning methods have similar accuracy. When using 10 power traces, the success rate is approximately around 70%. In terms of training time, SVM is the fastest, while LSTM takes the longest.

### The generality of distributed power analysis attack methods

This paper applies the method to the cracking of AES encryption chips, taking SVM as an example. Similarly, we selected 10,000 power traces as the training set and used the output values of the S-box as the intermediate values for the attack. We compared the training time and attack success rate of the two, and the results are shown in Table 5.

**Table 5:** Comparison of AES and SM4 encryption crack.

Encryption Method	Training time(S)	Success rate of cracking(used traces is below)			
		5	10	15	20
SM4	190	43%	72%	79%	87%
AES	107	55%	82%	87%	95%

From Table 5, it can be observed that the speed of cracking AES encryption chips is faster, and the success rate is higher. This is because in the AES encryption process, the key is XORed directly with the plaintext, making the key more vulnerable to exposure. On the other hand, SM4 generates round keys from the key before interacting with the plaintext, making it more concealed and challenging to crack.

### Attacking of masked SM4 encryption chip

In engineering, it is common to incorporate masking techniques into the encryption process to counteract side-channel attacks, such as power analysis attacks. Compared to standard encryption methods, masking involves operations like Galois multiplicate or XOR with intermediate values within the encryption process.

The location at which the sub-plaintext and the round key first interact during the SM4 encryption process:

24 $$\begin{aligned} V=X_1\oplus X_2\oplus X_3\oplus rk_0 \end{aligned}$$

If the mask is added before this point, it is treated as the key, and the method mentioned earlier is used to find the intermediate value, and the mask is attacked. If the mask is added after this point, the round keys are cracked first, and the entire mask and key are cracked through multiple iterations using this method.

In this paper, an attempt was made to perform XOR operations with a fixed mask at each S-box output, and using this method, all the keys and masks were successfully cracked.

## Cracking key from one power trace

### Attack principle

Building on the previous work, we shifted from classifying based on Hamming weight to classifying directly based on the key, turning a 9-class problem into a 256-class problem. Due to this finer classification, there was a noticeable decrease in classification accuracy, with the accuracy for each 8-bit key dropping from 98

**Figure 8:**
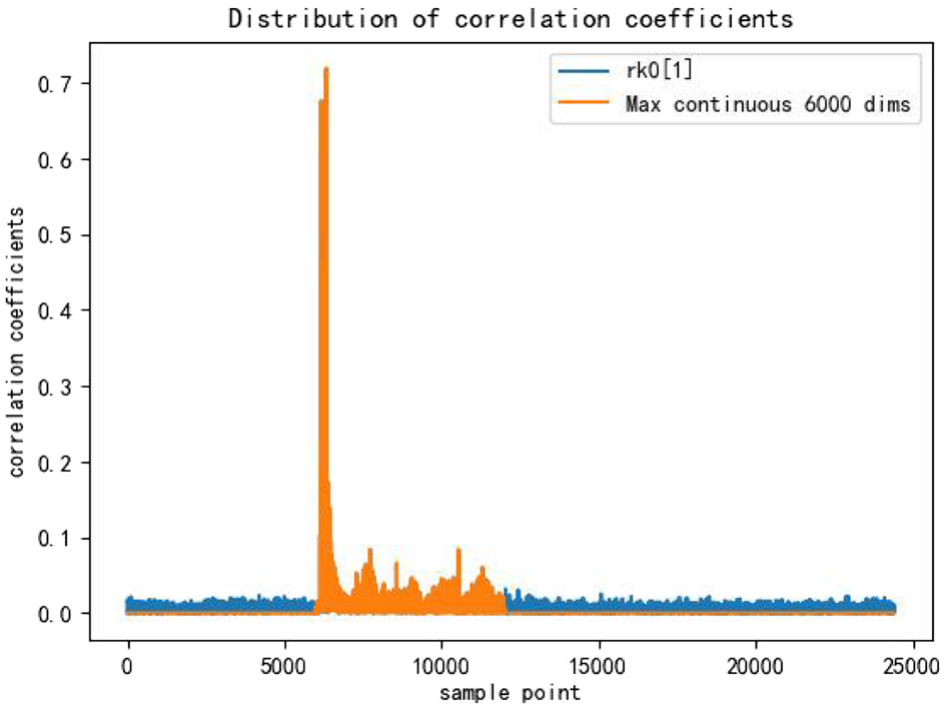
Max continuous 6000 dims.

1. As observed from Fig. 8, every 8-byte key is related to approximately 6000 sample points in the power trace. We chose the dimensionality of the features to be 6000.
2. To preserve the temporal characteristics, we selected a continuous window of 6000 sample points with the highest correlation coefficient.
3. We employed a 1D CNN+LSTM model for training.

These optimizations were implemented to improve the classification accuracy.

### Model establishment

**Figure 9:**
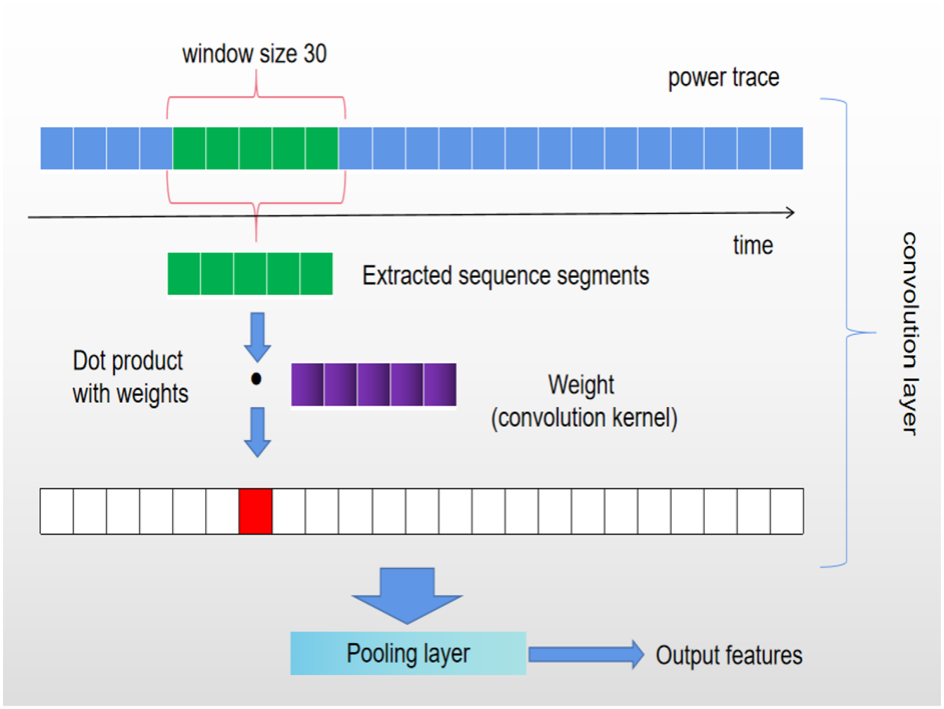
One-dim CNN.

Convolution and Pooling in 1D CNN (Fig. 9)

After multiple rounds of convolution and pooling, the data dimension is reduced from 6000 to 1000, and then a LSTM network is used for 256-class classification. The layer sizes are as follows: 1000$\rightarrow$800$\rightarrow$600$\rightarrow$400$\rightarrow$256.

### Attack performance

**Figure 10:**
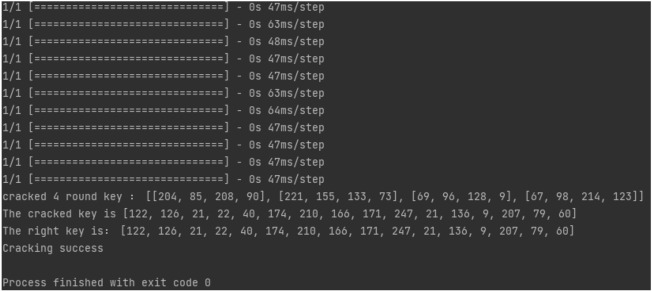
Cracking key from one power trace.

Due to the larger size of the model, training times often reach up to 5 h (without GPU). The classification success rate for 8-bit keys averaged at 92%. In the case of only 1 power trace available, the probability of successfully recovering all 16 roundkeys reached 28% (Fig. 10). With just 3 power traces available, the success rate for key recovery reached 45%

**Figure 11:**
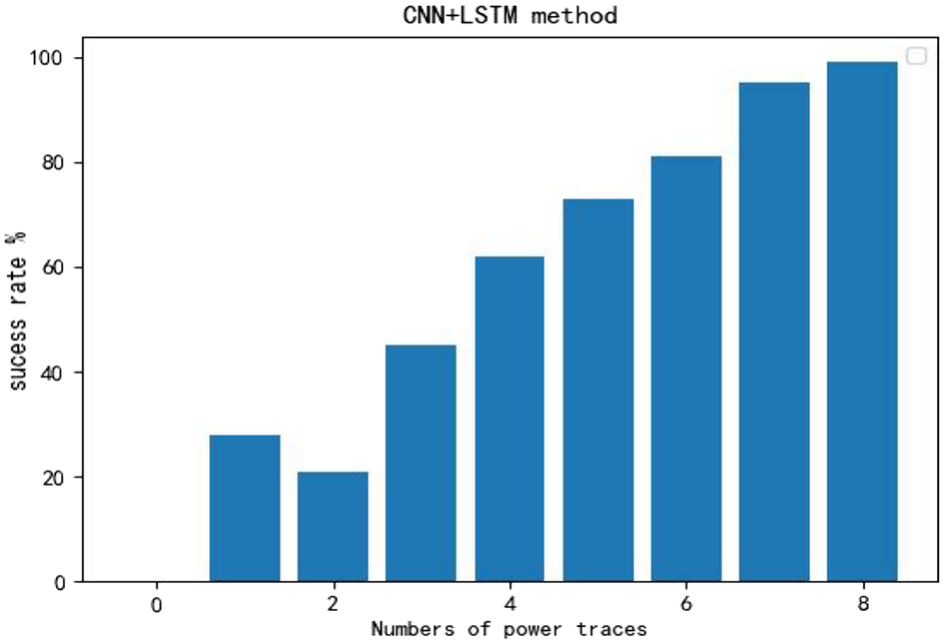
The success rate.

From Fig. 11, it can be observed that since it’s based on individual selections where each trace produces an independent result, having 2 traces doesn’t offer any improvement compared to just 1 trace. It randomly selects one result among them. When there are 8 traces available, the success rate for decryption reaches 99%.

## Conclusion

This paper has accomplished three main parts: the hardware implementation of the SM4 encryption algorithm, the collection of power traces, and the decryption of keys using power analysis attacks. In terms of key decryption, both common CPA methods and machine learning-based approaches have been employed. Additionally, a distributed decryption method has been utilized to enhance decryption speed and accuracy. With only 10 power traces, the success rate of decryption has been increased to over 70%, and the decryption time has been reduced by 76%. By using a 256-classification model, the success rate of decrypting a 128-bit key has reached 28% with only one power trace. Furthermore, this study has successfully broken the SM4 encryption chip with simple masking.

In real-world scenarios, encryption chips may perform other operations simultaneously during encryption, introducing noise that makes it difficult to capture power leakage, thereby increasing the difficulty of decryption. Additionally, the experiments in this paper heavily rely on clock alignment. Therefore, future research should focus more on noise filtering techniques to make power analysis attacks effective even in more complex situations.

## Data Availability

The datasets generated and analysed during the current study are available in the sm4_traces repository: https://drive.google.com/file/d/1dbUEeosEiT0eZ-31x-SC0aDxxT2UbQ-p/view?usp=sharing. SM4-cracker:https://drive.google.com/file/d/10wvZBfJDU1uf9VSLpVRcwiwy35n6OOrG/view?usp=sharing. All data generated or analysed during this study are included in this published article and its supplementary information files.

## Appendix

SM4 power traces download address:

https://drive.google.com/file/d/1dbUEeosEiT0eZ-31x-SC0aDxxT2UbQ-p/view?usp=sharing.

self-made SM4-cracker download address:

https://drive.google.com/file/d/10wvZBfJDU1uf9VSLpVRcwiwy35n6OOrG/view?usp=sharing.
